# BioTM Buzz (Volume 6, Issue 2)

**DOI:** 10.1002/btm2.10224

**Published:** 2021-05-08

**Authors:** Aaron C. Anselmo

**Affiliations:** ^1^ Division of Pharmacoengineering and Molecular Pharmaceutics Eshelman School of Pharmacy, University of North Carolina at Chapel Hill Chapel Hill North Carolina USA

## 1 IMPROVING QUANTITATIVE BIODISTRIBUTION ANALYSIS

Quantitative biodistribution measurements can provide essential information related to the potential toxicity and efficacy of drugs and drug delivery systems. Because of this, accurate biodistribution data can be used to inform the design and implementation of delivery systems that necessitate high levels of targeting, or low levels of off‐site accumulation. While there exist highly sensitive approaches to measure biodistribution (e.g., radiotracing), biodegradable delivery systems present a unique challenge as radiolabels can be unpredictably released during delivery system storage or biodegradation, which can lead to inaccurate biodistribution measurements. In this issue of *Bioengineering & Translational Medicine*, a team led by Professor Silvia Muro from the Institute for Bioscience and Biotechnology Research at the University of Maryland, College Park, and the Institute for Bioengineering of Catalonia of the Barcelona Institute of Science and Technology, describes an approach to separate unbound radiolabels from polymer‐bound radiolabels so as to accurately quantify the biodistribution of a DNA‐based delivery system termed 3DNA. It was demonstrated that ^125^I radiolabels were released from ^125^I‐labeled‐3DNA carriers upon introduction into biological fluids or tissues, thus demonstrating the need to separate the signals from the released radiolabel and the still‐carrier‐bound radiolabel. The authors then show that through precipitation initiated with trichloroacetic acid, free radiolabel could be quantified separately from the radiolabeled carrier. Importantly, biodistribution of ^125^I‐labeled‐3DNA could not be accurately quantified by simply considering the biodistribution of a separate control with free ^125^I radiolabel; instead, to accurately (>85% accuracy) quantify biodistribution of ^125^I‐labeled‐3DNA, the free ^125^I radiolabel must be precipitated out of each organ for each individual experiment. Overall, this study describes an important step in accurately quantifying the biodistribution of radiolabeled, biodegradable, delivery systems.

DOI: 10.1002/btm2.10208

## 2 IONIZABLE LIPID NANOPARTICLES FOR NUCLEIC ACID DELIVERY

Ionizable lipid nanoparticles have received increasing attention for their key role in facilitating the FDA approval of the first small interfering RNA (siRNA) therapeutic and most recently for their use in the lipid‐based nanoparticle SARS‐nCoV2 vaccines. Still, it is widely believed that the potential of ionizable lipid nanoparticles exceeds their demonstrated impact in these clinically used therapeutics. However, to achieve this potential, challenges related to their endosomal escape and subsequent delivery of nucleic acids to the cytosol must be addressed. In this article, authors from the labs of Dr. Paolo Decuzzi of the Fondazione Istituto Italiano di Tecnologia and Dr. Dan Peer of Tel Aviv University comprehensively review the history of ionizable lipid nanoparticles, highlight the challenges of intracellular delivery, and describe the known mechanisms by which endosomal escape can be achieved. Finally, the authors discuss how viruses, and their abilities to negotiate and breach the same barriers that limit lipid nanoparticle delivery systems, can potentially be used as a source of inspiration for the design of next‐generation lipid nanoparticles that can more‐readily escape the endosome.

DOI: 10.1002/btm2.10213

## 3 NANOPARTICLES THAT TARGET THE VASCULATURE: USING MICROPARTICLES THAT MARGINATE

A recent article from the lab of Professor Eniola‐Adefeso from the Department of Chemical Engineering at the University of Michigan, Ann Arbor, describes a novel approach to utilize the enhanced blood‐vessel margination of microparticles to increase the deposition and accumulation of nanoparticles to the vascular wall. In this manuscript, the authors synthesize a variety of nanoparticle‐loaded microparticles to evaluate the potential of using these marginating microparticles as a carrier for the vascular deposition of nanoparticles. The authors show that their microparticle‐mediated approach, which was targeted to the vascular endothelium via anti‐ICAM‐1, could lead to a >30‐fold increase as compared to anti‐ICAM‐1‐targeted nanoparticles. Overall, this article demonstrates a new approach to deliver nanoparticles directly to the vascular endothelium.

*Fish, M. B., Banka, A. L., Braunreuther, M., Fromen, C. A., Kelley, W. J., Lee, J., Adili, R., Holinstat, M., and Enioloa‐Adefeso, O. Deformable microparticles for shuttling nanoparticles to the vascular wall*. Science Advances *(2021), 7(17), eabe0143; DOI: 10.1126/sciadv.abe0143*

